# Fluoride and Arsenic Exposure Impairs Learning and Memory and Decreases mGluR5 Expression in the Hippocampus and Cortex in Rats

**DOI:** 10.1371/journal.pone.0096041

**Published:** 2014-04-23

**Authors:** Shoufang Jiang, Jing Su, Sanqiao Yao, Yanshu Zhang, Fuyuan Cao, Fei Wang, Huihui Wang, Jun Li, Shuhua Xi

**Affiliations:** 1 Department of Occupational and Environmental Health, Liaoning Provincial Key Lab of Arsenic Biological Effect and Poisoning, School of Public Health, China Medical University, Shenyang, Liaoning, P. R. China; 2 Department of Occupational and Environmental Health, Hebei Province Key Laboratory of Occupational Health and Safety for Coal Industry, School of Public Health, Hebei United University, Tangshan, Hebei, P. R. China; 3 Laboratory Animal Center, Hebei United University, Tangshan, Hebei, P. R. China; University of Missouri-Kansas City, United States of America

## Abstract

Fluoride and arsenic are two common inorganic contaminants in drinking water that are associated with impairment in child development and retarded intelligence. The present study was conducted to explore the effects on spatial learning, memory, glutamate levels, and group I metabotropic glutamate receptors (mGluRs) expression in the hippocampus and cortex after subchronic exposure to fluoride, arsenic, and a fluoride and arsenic combination in rats. Weaned male Sprague-Dawley rats were assigned to four groups. The control rats drank tap water. Rats in the three exposure groups drank water with sodium fluoride (120 mg/L), sodium arsenite (70 mg/L), and a sodium fluoride (120 mg/L) and sodium arsenite (70 mg/L) combination for 3 months. Spatial learning and memory was measured in Morris water maze. mGluR1 and mGluR5 mRNA and protein expression in the hippocampus and cortex was detected using RT-PCR and Western blot, respectively. Compared with controls, learning and memory ability declined in rats that were exposed to fluoride and arsenic both alone and combined. Combined fluoride and arsenic exposure did not have a more pronounced effect on spatial learning and memory compared with arsenic and fluoride exposure alone. Compared with controls, glutamate levels decreased in the hippocampus and cortex of rats exposed to fluoride and combined fluoride and arsenic, and in cortex of arsenic-exposed rats. mGluR5 mRNA and protein expressions in the hippocampus and mGluR5 protein expression in the cortex decreased in rats exposed to arsenic alone. Interestingly, compared with fluoride and arsenic exposure alone, fluoride and arsenic combination decreased mGluR5 mRNA expression in the cortex and protein expression in the hippocampus, suggesting a synergistic effect of fluoride and arsenic. These data indicate that fluoride and arsenic, either alone or combined, can decrease learning and memory ability in rats. The mechanism may be associated with changes of glutamate level and mGluR5 expression in cortex and hippocampus.

## Introduction

Fluoride (F) and arsenic (As) have been recognized worldwide as two serious inorganic contaminants in drinking water. Several areas throughout the world have both fluoride and arsenic present at high concentrations in ground water because of similar geogeny. Concurrent chronic poisoning caused by these two trace elements is an emergent endemic problem in many countries, including China, India, Mexico, Bangladesh, and Argentina [1]. In China, the prevalence of both endemic fluorosis and arseniasis is very serious, resulting especially from drinking water that contains high concentration of fluoride and arsenic. Concurrent chronic poisoning, referred to as a syndrome of endemic arsenism and fluorosis [2], mainly exists in certain places in China, such as Shanxi, Inner Mongolia, Xinjiang, Ningxia, and Qinghai provinces, because of high concentrations of fluoride and arsenic in drinking water. In some areas, this poisoning is caused by polluted air and food during coal burning, such as in Guizhou and Shaanxi. Additionally, the wide use of fluoride-containing toothpastes and mouth washes together with high arsenic in groundwater causes co-exposure to fluoride and arsenic.

Combined exposure to fluoride and arsenic may result in more complicated adverse health effects than exposure to fluoride or arsenic alone. Many health hazards caused by fluoride and arsenic alone have been reported [3]–[7], but less has been reported about their joint effects on health. Furthermore, some results have been contradictory. For example, different joint actions, including independent, synergistic, and antagonistic effects, have been observed in different experimental studies [8]–[12]. Therefore, further research is needed to reveal the interaction between fluoride and arsenic with regard to their toxic effects.

Fluoride and arsenic are able to cross the blood-brain barrier or placental barrier and accumulate in the brain in rats and mice exposed to high concentrations of fluoride and arsenic [13]–[18]. Some epidemiological studies have demonstrated that chronic exposure to fluoride [19]–[22] and arsenic [19], [22]–[27] in drinking water is associated with lower child intelligence and impairments in cognitive and neurobehavioral function. However, relatively little information is available in the literatures about the association between fluoride-arsenic co-exposure and cognitive and neurobehavioral function in humans. Both high arsenic and high fluoride exposure from drinking water were reported to impair child intelligence, but arsenic was shown to have more significant effects than fluoride [22]. Arsenic remains the primary factor that negatively affects child intelligence when fluoride and arsenic co-exist in drinking water. With regard to the interactive effect of fluoride and arsenic on learning and memory when administered concomitantly, few experimental data are available.

The above research indicates that fluoride and arsenic exposure alone can impair child intelligence and impair neurobehavioral function, including learning and memory. However, the molecular mechanisms of fluoride- and arsenic-induced learning and memory impairments have not been clearly defined. Individual fluoride or arsenic exposure can alter the levels of some neurotransmitters and activity of neurotransmitter metabolism-related enzymes, such as cholinesterase and dopamine β-hydroxylase, in the brain [28]–[35]. Nevertheless, relatively little is known about the effects of fluoride and arsenic on the glutamate neurotransmitter system in the hippocampus and cortex. Glutamate (glu), as a neurotransmitter, is the majority in excitatory synapses of the mammalian central nervous system, and it plays an important role through ionotropic and metabotropic glutamate receptors (mGluRs) in spatial learning and memory processes [36]–[38]. The role of mGluRs in synaptic transmission and synaptic plasticity, considered to be the basis of learning and memory, has been recently studied and characterized in the hippocampus [39]. mGluRs belong to the G-protein-coupled receptor (GPCR) superfamily and are subclassified into three groups with eight subtypes based on sequence homology, G-protein coupling, and ligand selectivity. Group I mGluRs include mGluR1 and mGluR5, both of which are abundant in the adult hippocampus and cerebral cortex, which are critical regions in the brain for learning and memory processes [40], [41]. They are also involved in the maintenance of synaptic plasticity [42]–[44]. mGluR5 mRNA and protein expressions decreased dose-dependently after lead exposure, suggesting that mGluR5s might be involved in lead-induced neurotoxicity [45]. However, in our knowledge, no studies have reported the effects of mGluR5s on learning and memory impairment induced by fluoride and arsenic.

In the present study, an *in vivo* rat model was used to determine whether fluoride and arsenic co-exposure reduces the spatial learning and memory ability. The mechanism was also explored by assessing the effects of fluoride, arsenic, and a fluoride + arsenic combination on mGluR5 mRNA and protein expressions in the hippocampus and cortex.

## Materials and Methods

### Chemicals

Sodium arsenite (NaAsO_2_, >99% purity) and sodium fluoride (NaF, >99% purity) were purchased from Sigma-Aldrich (St. Louis, MO, USA) and Beijing Chemical Reagent Corp. (Beijing, China), respectively. The two chemicals were dissolved in drinking water. The NaAsO_2_ solution was freshly prepared each day. All of the other chemicals used for hydride atomic fluorescence spectrophotometry and microplate reader-fluoride reagent spectrophotometry were analytical-grade. The standard reference material, iAs III (GBW 08611), was obtained from the National Center for Standard Reference Materials (Beijing, China), containing 1 mg/ml iAs III. Fluoride standard solutions of 1.000 and 10.00 µg/ml were obtained from Beijing Beihua Fine Chemicals Co. Ltd (Beijing, China).

### Experimental Animals and Treatments

A total of 56 weaned specific-pathogen-free (SPF) male Sprague-Dawley rats (60–80 g) were kept in a standard animal house with an ambient temperature of 25±1°C and relative humidity of 50±5% under a 12 h/12 h light/dark cycle. The rats were given standardized pellet food and water *ad libitum*. After 1 week of adaptive feeding, the rats were randomly assigned to a control group, fluoride (F) group, arsenic (As) group, and fluoride + arsenic (F+As) group (*n* = 14 per group). The rats in the F, As, and F+As groups had access to drinking water with a 120 mg/L NaF solution, 70 mg/L NaAsO_2_ solution, and combined 120 mg/L NaF and 70 mg/L NaAsO_2_ solution for 3 months, respectively. The control rats were given drinking water for 3 months. The body weights of all of the rats were recorded every 7 days, and the volume of water consumed was measured daily.

After 3-month exposure, eight rats (selected randomly) from each group were tested in the Morris water maze (MWM). After the MWM test was completed, the rats were intraperitoneally anesthetized with 10% chloral hydrate. Blood was collected by heart puncture. The hippocampus and cerebral cortex were quickly separated on ice, immediately transferred to liquid nitrogen, and stored at −80°C until analysis.

### Ethics Statement

This study was performed in accordance with the recommendations of the Guide for the Care and Use of Laboratory Animals of the China National Institute of Health. The protocol was approved by the Committee of Hebei United University (permit no. 2011-002). All of the surgeries were performed under 10% chloral hydrate anesthesia, and all efforts were made to minimize suffering.

### Detection of Fluoride and Arsenic Concentrations

The concentrations of fluoride in serum and the hippocampal and cortical tissue samples were measured using an enzyme standard instrument-fluorine reagent colorimetric method described in detail by Zhang et al. [46]. The concentrations of total arsenic in whole blood and the hippocampal and cortical tissue samples were detected using hydride atomic fluorescence spectrophotometry according to Luo et al. [17]. The results are expressed as micrograms per liter (blood) or micrograms per gram of wet tissue weight.

### Evaluation of Spatial Learning and Memory in the Morris Water Maze

The training and testing of spatial learning and memory ability were performed in an SLY-WMS MWM automatic control and recording system (Beijing Sunny Instruments Co. Ltd.). The general characteristics of the apparatus, including size, composition, and locations of the quadrants and platform, were described in detail by Fan et al. [47]. Spatial learning and memory function in rats was assessed in the MWM by conducting two different tests-hidden platform acquisition and probe trial test-according to Fan et al. [47] and Luo et al. [17] with some modifications.

In the hidden platform acquisition test, the rats underwent 4 consecutive days of training in the MWM that was filled with tap water (25±1°C) with two trials each day. In each trial, the rats were released from four randomly assigned release points (N, W, S, and E). The rats were placed in the tank, facing the wall of the pool, and allowed to freely swim to search for the escape platform for a maximum of 120 s. The time to reach the platform was recorded as the escape latency. The rats were permitted to rest on the platform for 30 s before the next trial. If a rat failed to find the platform within 120 s, then it was guided to the platform by a stick and placed on the platform for 30 s; in this case, the escape latency was recorded as 120 s for this trial.

After the hidden platform acquisition test, a probe test was conducted by removing the platform. The rats were allowed to freely swim in the MWM for 120 s. The time spent in the target quadrant where the hidden platform was previously located, the time of first crossing the location where the platform was originally located, and the numbers of the rat passed through the original hidden platform location were recorded. The three indices, especially the time spent in the target quadrant, indicate the degree of memory maintenance and consolidation.

After the 4-day training period, the rats were returned to their cages and allowed to rest for 1 week. These trained rats were then placed in the MWM again and tested under the same conditions to evaluate long-term memory ability. In this test phase, the rats completed hidden platform acquisition and the probe test without training.

### Determination of Glutamate Neurotransmitter Level

The homogenate of the hippocampal and cortical tissue was centrifuged for 15 min at 2,000 rotations per minute (rpm), and the supernatant together with serum was used for glutamate determination. Glutamate was measured using a reagent kit (Nanjing Jiancheng Bioengineering Institute, Nanjing, China) by a microplate reader (VersaMax, Molecular Devices, Sunnyvale, CA, USA) at 340 nm. Glutamate levels in serum and supernatant were calculated according to the standard curve of the kits.

### Reverse Transcriptase-polymerase Chain Reaction

Total RNA in the hippocampal and cortical tissue was isolated using Trizol reagents (Invitrogen, Carlsbad, CA, USA) and dissolved in RNase-free water. For each sample, 2 µg of total RNA was converted into first-strand cDNA using the One-Step RT-PCR SuperMix kit (TransGen Biotech, Beijing, China) according to the manufacturer’s recommended protocol. Specific primers for the target transcripts were designed (Takara Biotechnology Co., Ltd) and are listed in Table 1. Reverse transcription-polymerase chain reaction (RT-PCR) was performed using 25 µl mixing reaction buffer with 1 µl RNA, gene-specific primers at a final concentration of 0.2 µM, and enzymes according to the instructions from the manufacturer. The PCR program for mGluR1 was the following: 94°C for 5 min, followed by 28 cycles (94°C for 30 s, 58°C for 30 s, and 72°C for 30 s), with final extension at 72°C for 7 min. The PCR program for mGluR5 was the following: 94°C for 5 min, followed by 28 cycles (94°C for 30 s, 58°C for 30 s, and 72°C for 1 min), with final extension at 72°C for 5 min. Reactions that omitted DNA polymerase were used as a control for contamination. The PCR products were run on 2% agarose gel and stained with ethidium bromide, and photographs were taken under ultraviolet light. The band intensities were quantified using Quantity One software (Bio-Rad, Hercules, CA, USA). The relative level of mRNA expression was normalized to β-actin.

**Table 1:** Gene-specific primer sets and PCR parameters.

Gene name	Primer sequences		Cycles	Product (bp)
mGluR1	Sense	5′-CATCCCACAGATCGCCTATT-3′	28	100
	Anti-sense	5′-TGCCTGCAAAGTGTCAGAAG-3′		
mGluR5	Sense	5′-CACTCTTGCCCAACATCAC-3′	28	138
	Anti-sense	5′-CACAGCGTACCAAACCTTC-3′		
β-actin	Sense	5′-AGCCATGTACGTAGCCATCC-3′	30	115
	Anti-sense	5′-ACCCTCATAGATGGGCACAG-3′		

### Western Blot

The hippocampal and cortical tissues were homogenized and incubated at 4°C overnight in a RIPA lysis buffer solution (Beyotime Institute of Biotechnology, Haimen, China) that contained protease and phosphatase inhibitors (Sigma-Aldrich, St. Louis, MO, USA). These samples were centrifuged at 12,000×*g* for 1 h at 4°C, and the supernatant was then separated. Total protein concentrations were determined using a BCA protein assay kit (Santa Cruz Biotechnology, Santa Cruz, CA, USA). An equal amount of protein (50 µg) for each sample was separated by 10% sodium dodecyl sulfate-polyacrylamide gel electrophoresis (SDS-PAGE) and then transferred to a polyvinylidene difluoride (PVDF) membrane (Millipore Corporation, Billerica, MA, USA) using a transfer unit (Bio-Rad, Hercules, CA, USA). After being blocked with 5% milk and washed with TTBS (0.1% Tween 20, 100 mM Tris-HCl, and 0.9% NaCl), the PVDF membrane was incubated with rabbit polyclonal anti-mGluR5 antibody (1∶1000 dilution; Anbo Biotechnology, San Francisco, CA, USA) at 4°C overnight. After washing, the membrane was incubated with horseradish peroxidase-conjugated anti-rabbit immunoglobulin G (1∶5000 dilution; Santa Cruz Biotechnology, Santa Cruz, CA, USA) at room temperature for 1 h. Finally, the membrane was incubated in ECL Plus reagent (PerkinElmer, Waltham, MA, USA) for 5 min, and the signals were visualized using an AlphaImager system analyzer (ProteinSimple, Santa Clara, CA, USA). The immunoblot densities were calculated using an AlphaImager system analyzer. The relative level of protein expression was normalized to β-actin.

### Statistical Analysis

The data are expressed as mean ± standard deviation (SD), and statistical significance was determined using one-way analysis of variance (ANOVA), followed by the Least Significant Difference (LSD) *post hoc* test. Alterations in daily water intake, body weights of rats with exposure time and escape latency with training days were analyzed using repeated measures design analysis of variance. All of the statistical analyses were two-sided (α = 0.05) and performed using SPSS 17.0 software (SPSS, Chicago, IL, USA).

## Results

### Growth and Development

**Figure 1:**
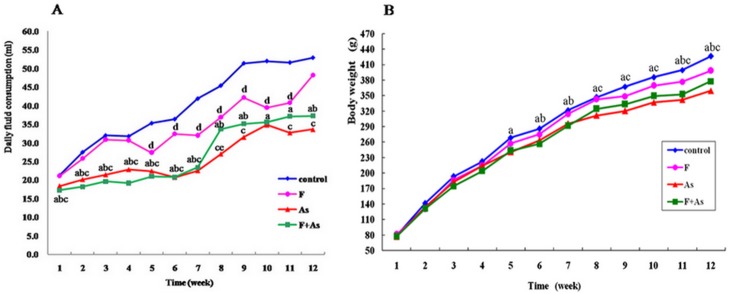
Effects of fluoride, arsenic, and fluoride + arsenic on body weights and daily fluid consumption in rats. Data represent mean for all animals of each group (*n* = 14). ^a^ *p*<0.05, arsenic group and fluoride + arsenic group vs. control group, respectively;^ b^ *p*<0.05, fluoride + arsenic group vs. fluoride group; ^c^ *p*<0.05, arsenic group vs. fluoride group;^ d^ *p*<0.05, fluoride group vs. control group;^ e^ *p*<0.05, fluoride + arsenic group vs. arsenic group.

All rats were observed once daily with detailed evaluation on general appearance and physical condition including moving activities, appetite, appearance of hair, nose, eyes and limbs, and no obvious change was noticed. There was no mortality in rats of any groups. The average daily water consumption of rats every week is shown in Fig. 1A. Repeated measures with treatment as between-subjects factor and exposure time as within-subjects variables showed a significant treatment effect (*F* = 173.84, *p*<0.01), a significant exposure time effect (*F* = 159.21, *p*<0.01), and an interaction between treatment and exposure time (*F* = 5.22, *p*<0.01). The daily water consumption of rats in F group was less than the control group from week 5 to week 11. The daily water intake of rats in As and F+As group was all less than the control and F group. From the 7th week to the end of the study, the mean daily water consumption of rats in As group was lowest.

As shown in Fig. 1B, the body weights of rats in four groups all increased with exposure time extended. There were significant effects of treatment (*F* = 20.69, *p*<0.01) and exposure time (*F* = 636.72, *p*<0.01), but no interaction between treatment and exposure time (*F* = 1.00, *p*>0.05). Compared with control group, rats in As group and F+As group gained less body weights from week 5 to the end of the experimental period. After the 8th week of exposure, difference of the body weights of rats among four groups was more obvious. From week 8 to the end of the study, the mean body weight of rats in As group was lowest, and it was not only lower than controls, but also than F group. On the 6th, 7th, 11th and 12th week, the mean body weight of rats in F+As group was also lower than F group (*p*<0.05). Rats exposed to fluoride did not show significant difference in body weight compared with control rats during the whole experimental period.

### Fluoride and Arsenic Concentrations in Blood, the Hippocampus and the Cortex

The levels of fluoride in serum, the hippocampus and the cortex in 120 mg/L NaF-treated rats (F group and F+As group) increased significantly compared with the non-fluoride-treated group (control and As groups; all *p*<0.01). The level of fluoride was the same in the F group and F+As group. The levels of arsenic in whole blood, the hippocampus and the cortex in 70 mg/L NaAsO_2_-treated rats (As group and F+As group) were much higher than in the control and F groups (all *p*<0.01). The level of arsenic was the same in the As group and F+As group. No significant differences in fluoride or arsenic content were found between the hippocampus and cerebral cortex (*p*>0.05; Table 2).

**Table 2:** Fluoride and arsenic content in blood, the hippocampus, and the cortex (mean ± SD; *n* = 6).

Groups	Blood (µg/ml)		Hippocampus (µg/g)			Cortex (µg/g)		
	Fluoride*	Arsenic#	Fluoride		Arsenic	Fluoride	Arsenic	
Control	0.09±0.06	25.60±3.73	0.24±0.18	0.16±0.03		0.25±0.07	0.14±0.02	
F group	0.40±0.07^ab^	23.33±3.70	1.14±0.35^ab^	0.16±0.02		1.19±0.42^ab^	0.16±0.03	
As group	0.09±0.04	200.02±34.07^ac^	0.48±0.10	3.92±0.63^ac^		0.41±0.03	3.73±0.99^ac^	
F+As group	0.44±0.13^ab^	211.40±14.13^ac^	1.06±0.27^ab^	3.80±0.76^ac^		1.03±0.21^ab^	3.43±0.63^ac^	
*F*	41.52	184.63	19.24	64.42		22.49	47.07	
*p*	<0.001	<0.001	<0.001	<0.001		<0.001	<0.001	

*Fluoride content in serum in rats.

# Arsenic content in whole blood in rats.

a *p*<0.01, compared with control group.

b *p*<0.01, compared with As group.

c *p*<0.01, compared with F group.

### Spatial Learning and Memory Ability

**Figure 2:**
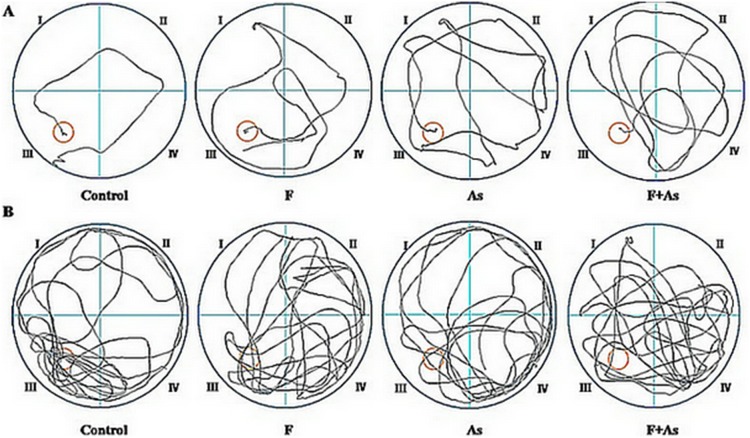
Representative swimming tracks. A) Tracks of finding the hidden platform in rats on the fourth day of training in the navigation test. The diagram of the tracks shows that the control rats more easily found the platform, whereas the rats in the fluoride, arsenic, and fluoride + arsenic groups found the platform through repeated exploration. Red circle: hidden platform; black line: swimming tracks. B) Tracking pictures in the 120 s probe trial test on the fourth day in rats. The diagram of the tracks indicate that the control rats repeatedly passed through the region where the original hidden platform was located, whereas the rats in the fluoride, arsenic, and fluoride + arsenic groups passed through the region of the platform only a few times.

The average escape latency in rats is shown in Table 3 and Fig. 2A. In the MWM hidden platform acquisition test, as the training days increased, the average escape latencies in four groups showed a decreasing trend (*p*<0.05), indicating that the rats in each group were able to gain space allocation memory for the platform underwater. There were significant effects of treatment (*F* = 3.96, *p*<0.05) and training day (*F* = 28.36, *p*<0.01), but no interaction between treatment and training day (*F* = 0.47, *p*>0.05). Prolonged escape latency was observed in the F, As, and F+As groups compared with the control group from the first day to the fourth day of training. The average escape latency on the 11th day showed the same trend. No significant difference in escape latency was found among the F, As, and F+As groups.

**Table 3:** Escape latency (s) in rats in the navigation test in the Morris water maze (mean ± SD; *n* = 8).

Groups	First day	Second day	Third day	Fourth day	Eleventh day
Control	11.42±3.68	6.62±1.43	6.29±1.32	5.85±1.98	4.16±0.79
F group	19.03±4.08^a^	16.34±4.78^a^	12.31±7.14^a^	10.34±1.18^b^	14.92±1.80^a^
As group	19.06±5.80^a^	16.07±3.43^a^	14.39±3.27^a^	11.76±1.23^b^	14.83±2.10^a^
F+As group	24.65±6.98^a^	19.02±8.43^a^	15.00±2.90^a^	13.78±3.49^b^	17.36±3.50^a^
*F*	6.29	4.35	7.67	2.46	4.33
*p*	0.03	0.02	0.01	<0.001	0.013

a *p*<0.05, compared with control group.

b *p*<0.01, compared with control group.

The short-term and long-term memory data in the probe trial test are shown in Table 4 and Fig. 2B. A similar trend was observed on the fourth day and 11th day of the test. The time to find initially the platform in the three exposure groups was significantly longer compared with controls (all *p*<0.05). The time spent in the target quadrant and number of times that the rats passed through the original platform location within 120 s in the three exposure groups all decreased (all *p*<0.05) compared with control rats. No significant differences were observed in these indices among the F, As, and F+As groups.

**Table 4:** Spatial memory ability in rats in the probe trial test (mean ± SD; *n* = 8).

Groups	Fourth day			Eleventh day		
	Firstplatformtime (s)	Target quadranttime(s)	NumbersOfpassing	Firstplatformtime (s)	Target quadranttime (s)	Numbers of passing
Control	8.78±4.23	59.55±5.20	7.57±1.62	13.63±2.39	47.49±8.65	7.50±2.00
F group	15.67±3.13^b^	38.89±3.73^a^	5.57±1.62^a^	19.18±3.44^b^	37.81±9.13^a^	5.00±2.65^a^
As group	16.35±3.00^b^	38.31±8.90^a^	5.50±1.38^a^	19.95±2.95^b^	36.19±1.61^a^	4.50±1.76^a^
F+As group	17.81±3.39^b^	34.02±8.13^a^	3.86±1.77^a^	21.11±2.95^b^	32.12±6.46^a^	3.33±1.21^a^
*F*	10.83	10.22	2.65	42.54	11.12	6.75
*p*	<0.001	0.013	0.027	<0.001	0.016	0.002

a *p*<0.05, compared with control group.

b *p*<0.01, compared with control group.

### Glutamate Concentrations in Serum, the Hippocampus, and the Cerebral Cortex

**Figure 3:**
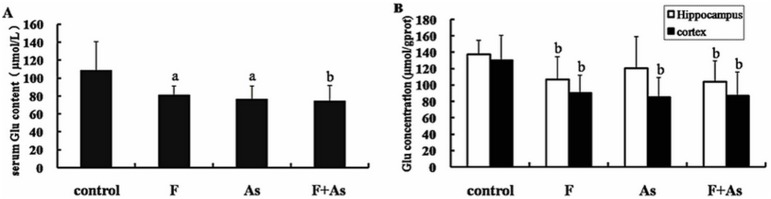
Effects of fluoride, arsenic, and fluoride + arsenic on glutamate concentrations in serum (A) and the hippocampus and cortex (B) in rats. The bars indicate the mean ± SD (*n* = 8). ^a^ *p*<0.05, ^b^ *p*<0.01, compared with the control rats.

As shown in Fig. 3, glutamate concentrations in serum, the hippocampus, and the cerebral cortex in the F and F+As groups were much lower than in the control group (*p*<0.05). Glutamate concentrations in serum and the cerebral cortex in the As groups were much lower than in the control group (*p*<0.05). No significant differences were observed in glutamate levels among the F, As, and F+As groups.

### mGluR1and mGluR5 mRNA Expression in the Hippocampus and Cerebral Cortex

**Figure 4:**
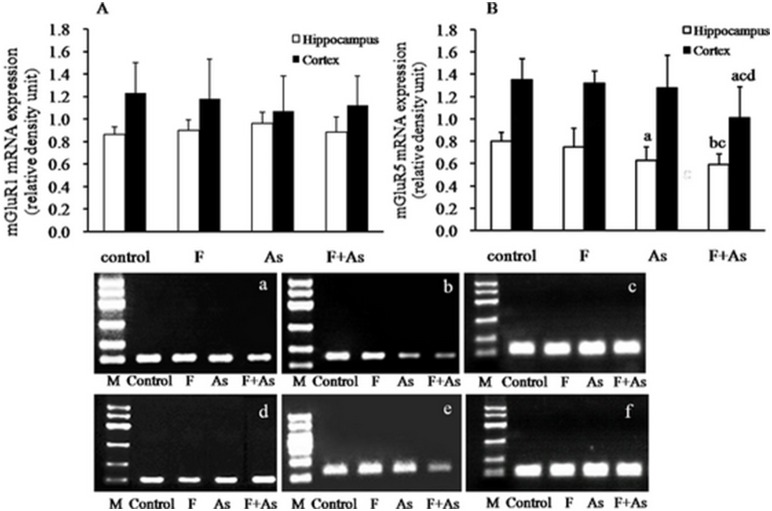
mGluR1 and mGluR5 mRNA expression in the hippocampus and cortex in rats. The bars indicate the mean ± SD (*n* = 8). ^a^ *p*<0.05, ^b^ *p*<0.01, compared with control rats;^ c^ *p*<0.05, compared with fluoride group; ^d^ *p*<0.05, compared with arsenic group. Panels a, b and c represent mGluR1, mGluR5 and β-actin mRNA expression in the hippocampus. Panels d, e and f represent mGluR1, mGluR5 and β-actin mRNA expression in the cortex. M: marker. The size from top to bottom is 600, 500, 400, 300, 200, and 100 bp, respectively. The PCR product for mGluR1, mGluR5 and β-actin was 100, 138 and 115 bp, respectively.

As shown in Fig. 4A, there were no differences in mGluR1 mRNA expression in the hippocampus and cortex among the four groups (*F* = 1.64, *p* = 0.201, and *F* = 0.47, *p* = 0.704, respectively). Significant differences were found in mGluR5 mRNA expression in the hippocampus and cortex among the four groups (*F* = 4.32, *p* = 0.013, and *F* = 3.48, *p* = 0.030, respectively). As shown in Fig. 4B, mGluR5 mRNA expression in the hippocampus in the As group and F+As group was much lower than the control rats (*p*<0.05 and *p*<0.01, respectively). Furthermore, the decrease of mGluR5 mRNA expression was also found in the F+As group compared with the F group. Compared with control rats, mGluR5 mRNA expression in the cortex decreased in the F group and As group, but the differences were not statistically significant. mGluR5 mRNA expression in the cortex in the F+As group was lower than in the control, F, and As groups (all *p*<0.05).

### mGluR5 Protein Expression in the Hippocampus and Cerebral Cortex

**Figure 5:**
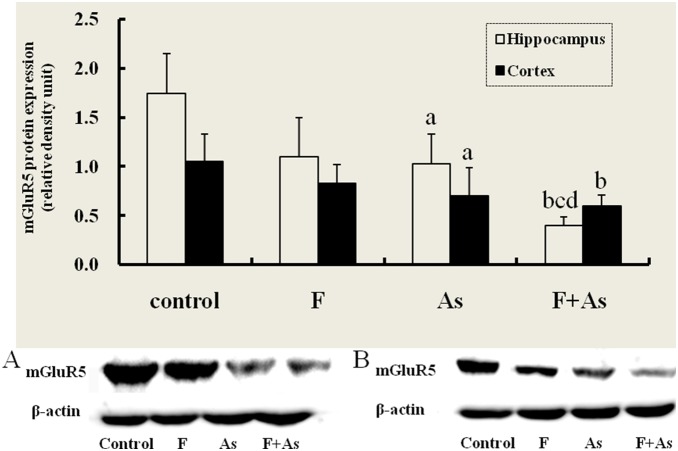
mGluR5 protein expression in the hippocampus and cortex in rats. The bars indicate the mean ± SD (*n* = 8). ^a^ *p*<0.05, ^b^ *p*<0.01, compared with control rats;^ c^ *p*<0.01, compared with fluoride group; ^d^ *p*<0.01, compared with arsenic group. Panels A and B represent mGluR5 and β-actin protein expression in the hippocampus and cortex, respectively.

mGluR5 mRNA expression in the hippocampus and cortex decreased in As and F+As group in this study. Furthermore, mGluR5 protein levels in the hippocampus and cortex were determined by Western blot (Fig. 5). There were significant differences in mGluR5 protein expression in the hippocampus and cortex among the four groups (*F* = 39.91, *p*<0.001, and *F* = 4.19, *p* = 0.019, respectively). A marked decrease in mGluR5 protein levels was found in the hippocampus in the As group and F+As group compared with the control group (*p*<0.05 and *p*<0.01, respectively). mGluR5 protein expression in the F+As group was also lower than in the F and As groups. Compared with control rats, mGluR5 protein levels in the cortex in the As group and F+As group decreased. Lower mGluR5 protein expression was observed in the hippocampus and cortex in the F group compared with control group, but the differences were not statistically significant.

## Discussion

In our preliminary experiments, we found that the mean daily water intake in 280–300 g rats was 30 ml. The LD_50_ of oral NaF and NaAsO_2_ in rats was 52 and 41 mg/kg, respectively. According to the average daily water intake, in the present study, rats that weighed 280–300 g consumed 12.0–12.8 mg/kg NaF per day and 7.0–7.5 mg/kg NaAsO_2_ per day. The results showed that fluoride and arsenic levels in blood, the hippocampus, and the cortex in rats exposed to fluoride (F and F+As groups) and arsenic (As and F+As groups) for three months were significantly higher than in non-exposed rats. These findings indicate that excessive fluoride and arsenic can cross the blood-brain barrier and accumulate in the brain in rats, which is consistent with previous studies [13]–[18], [48]. No significant differences in fluoride concentrations were found between the F group and F+As group. Similar results were observed for arsenic, suggesting no joint action of arsenic and fluoride with regard to accumulation in the brain in rats. However, Mittal et al. [9] reported that arsenic concentrations in the blood and liver in mice co-exposed to fluoride and arsenic decreased compared with arsenic exposure alone, whereas the arsenic concentration increased in the kidneys. The level of fluoride was the same in mice treated with fluoride alone and co-exposed to arsenic and fluoride. These results appear to be inconsistent with the present study, which may be attributable to the dose and duration of exposure and animal species. This discrepancy with regard to the interaction between arsenic and fluoride requires further investigation.

A significant decrease of body weight was observed in rats exposed to arsenic alone and co-exposed to fluoride and arsenic when compared with that of the control and fluoride-exposed rats. These results suggest that arsenic might have more significant effect on gain of body weight than fluoride in rats. This was consistent with previous study [49]. Body weight decreases found in rats treated with arsenic are in agreement with previous studies [14], [17], [50], [51]. In the present study there were no differences in food intake among four groups during the experimental period. So the loss of body weight caused by arsenic was not related to food intake, and could be a result of reduction in the repair and synthetic activities of various cells [52].

Fluoride and arsenic are neurotoxicants that can impair cognitive capacity. In the MWM hidden platform acquisition test, as the training days increased, the mean escape latencies in the four groups showed a decreasing trend, indicating that the rats in each group were able to find the hidden platform submerged below the surface of the water. There were no significant differences in the swimming velocity among the four groups, which suggested that the loss of body weight induced by arsenic in rats did not influence their performance in MWM test. Compared with control rats, the rats that were exposed to fluoride and arsenic alone exhibited obvious delays in finding the hidden platform not only on the four training days but also on the 11th day. These results suggest that fluoride and arsenic may impair spatial learning and memory ability. In the probe test on both the fourth and 11th days, the time to first find the initial platform location increased, and the time spent in the target quadrant and number of times that the rats passed through the original platform position within 120 s decreased in the F and As groups. These findings indicate that fluoride and arsenic may impair both short-term and long-term spatial memory in rats. Our results were consistent with other epidemiological and experimental animal studies, in which fluoride exposure was reported to be associated with decreased intelligence and learning and memory ability [19], [20], [22], [31], [53], [54]. Learning and memory ability in rat offspring also declined with exposure to high fluoride concentrations [48], [55]. Our results are also consistent with other published epidemiological data that intelligence quotient decreased in arsenic exposure children [19], [22]–[25] and learning and memory ability decreased in animals [14], [15], [17], [56], [57]. Notably, the rats exposed to either fluoride or arsenic in the present study exhibited similar escape latencies, a similar time to find the original platform location, a decrease in the time spent in the target quadrant, and a decrease in the number of times the rats passed through the original platform location. Although these changes were more serious in the rats of combined exposure to fluoride and arsenic than the rats of fluoride and arsenic exposure alone, no statistic differences were found in spatial learning and memory between the F or As group and F+As groups. At present, few studies have reported the association between fluoride and arsenic co-exposure and cognitive capacity. Wu et al. [35] reported that learning and memory ability decreased in rats individually exposed to fluoride and arsenic and in rats co-exposed to the two elements. Learning and memory ability in rat offspring co-exposed to fluoride and arsenic also decreased [58]. These findings are consistent with our results.

Glutamate is the major excitatory neurotransmitter in the mammalian central nervous system and plays an important role in spatial learning and memory function. In the present study, we found that fluoride or arsenic significantly reduced glutamate levels in the hippocampus and cortex in rats. A similar trend was observed in the F+As group. These findings imply that glutamate may be involved in learning and memory dysfunction induced by fluoride and arsenic exposure. Niu et al. (2009) [55] reported that fluoride decreased glutamate levels and changed the activity of glutamate metabolism-related enzymes, including aspartate aminotransferase, alanine aminotransferase, and glutamic acid decarboxylase, in the hippocampus in rats. These changes may be related to lower learning ability induced by fluoride in rats. Arsenic exposure was also reported to alter the activity and mRNA expression of glutamate metabolism-related enzymes in the brain in rat offspring, which may lead to neurobehavioral and learning and memory impairments [34]. In this study, glutamate levels in serum also decreased in fluoride or/and arsenic treated rats. In general, amino acid concentrations in brain are controlled by selective transport mechanisms at the blood-brain barrier and by specific metabolizing enzymes within the tissue. Brain concentrations of amino acids do not easily influence blood amino acid levels. Blood amino acid concentrations primarily reflect dietary composition, but also reflect changes in amino acid metabolism in many tissues throughout the body. Therefore, under the same feed, glutamate levels in serum may partly be influenced by the glutamate concentration in brain.

Several lines of evidence suggest that group I mGluRs play a critical role in regulating synaptic transmission and synaptic plasticity [59]. mGluR5, a subtype of group I mGluRs, is involved in the induction and maintenance of synaptic plasticity and formation of spatial learning and memory [42]–[44], [60], [61]. In rats, mGluR5 inhibition blocked spatial learning [62]. An allosteric mGluR5 potentiator markedly improved hippocampus-dependent spatial learning [63]. In the mice of lacked mGluR5, hippocampal long-term potentiation was abnormal, and the mice exhibited deficits in both spatial learning and fear conditioning [64], [65]. Our results showed that mGluR5 mRNA and protein expressions in the hippocampus and cortex in arsenic-exposed rats markedly decreased. However, no association was found between fluoride exposure and mGluR5 mRNA or protein expression in the hippocampus or cortex. These findings indicate that individual arsenic exposure and combined fluoride and arsenic exposure have more significant effects on mGluR5 expression than fluoride exposure alone. Interestingly, compared with fluoride or arsenic exposure alone, rats in the F+As group exhibited a downregulation of mGluR5 mRNA expression in the cortex and mGluR5 protein expression in the hippocampus. These results suggest that a synergistic interaction exists between these two trace elements. Altogether, mGluR5s may be involved in spatial learning and memory impairment caused by arsenic exposure and combined fluoride and arsenic exposure. In our study, there were no differences among four groups for mGluR1 mRNA expression. Although mGluR1 and mGluR5 are both G-protein-coupled mGluRs that stimulate intracellular Ca^2+^ release, they have distinct signaling properties and effects on neuronal function. Choi et al. (2011) [66] reported that calmodulin (CaM) is a strong regulator of mGluR5 trafficking and mGluR5-induced calcium signaling. Although it has been accepted that both mGluR1 and mGluR5 interact with CaM, they showed that CaM specifically binds mGluR5 and not mGluR1. Our study supported that fluoride and/or arsenic only changed the expression of mGluR5, not for mGluR1.

In conclusion, fluoride and arsenic can cross the blood-brain barrier and accumulate in the brain in rats. Fluoride and arsenic, either alone or combined, at the concentrations of 120 mg/L sodium fluoride and/or 70 mg/L sodium arsenite in drinking water, impaired spatial learning and memory ability and led to mild cognition impairment in rats. The reductions of glutamate levels and mGluR5 mRNA and protein expressions in the hippocampus and cortex may be involved in impairments in learning and memory ability.

## Supporting Information

File S1 **The animal experimental ethical inspection form and the guide for the care and use of Laboratory animals.**
(PDF)
