# Supplementary material for: Fluoride and Arsenic Exposure Impairs Learning and Memory and Decreases mGluR5 Expression in the Hippocampus and Cortex in Rats
Source: PLoS One. 2014 Apr 23;9(4):e96041. doi: 10.1371/journal.pone.0096041 (PMC3997496; doi:10.1371/journal.pone.0096041)
Supplement: File S1 — The animal experimental ethical inspection form and the guide for the care and use of Laboratory animals. (PDF) [file pone.0096041.s001.pdf]

河北联合大学动物实验伦理审查表

Animal Experimental Ethical Inspection Form of Hebei United University

编号（No）: 2011－002

<table>
<tr><td rowspan="12">申请人填写的相关信息 (Related information filled by applicant)</td><td colspan="2">申请单位
Name of organization</td><td colspan="3">公共卫生学院
School of Public Health</td></tr>
<tr><td colspan="2">经费来源
Funding source</td><td colspan="3">国家自然基金委员会
National Natural Science Foundation of China</td></tr>
<tr><td colspan="2">申请人
Applicant</td><td colspan="3">蒋守芳
Shoufang Jiang</td></tr>
<tr><td colspan="2">联系电话
Telephone</td><td colspan="3">0315-3725736</td></tr>
<tr><td colspan="2">申请日期
Application date</td><td colspan="3">2011 年 3 月 3 日</td></tr>
<tr><td colspan="2">实验名称
Experiment title</td><td colspan="3">氟砷联合暴露损害仔鼠学习记忆及Ⅰ组 mGluRs 介导的信号传导机制研究
Fluoride and arsenic co-exposure impair learning and memory and GroupⅠmGluRs mediated signal path mechanism in offspring rats</td></tr>
<tr><td colspan="2">拟实验时间
Experiment date</td><td colspan="3">2012 年 1 月 1 日至 2014 年 12 月 31 日</td></tr>
<tr><td colspan="2">实验设施合格证编号
Number of Experimental Facilities certification</td><td colspan="3">SYXK（冀）2010-0038</td></tr>
<tr><td rowspan="3">使用动物情况</td><td>动物来源
Source of animal</td><td>北京维通利华实验动物技术有限公司
Vital River Laboratory Animal Technology Co. Ltd.</td><td>质量合格证编号
Number of Quality certification</td><td>SCXK（京）2006-0009</td></tr>
<tr><td>品种品系
Species of strain</td><td>Sprague-Dawley rat</td><td>等级
Grade</td><td>SPF</td></tr>
<tr><td>数量
Number</td><td>♂ 120 只、♀ 120 只，共 240 只</td><td>规格
Specifications</td><td>21 天、成年鼠</td></tr>
<tr><td colspan="5">实验要点：包括实验目的、实验方法、观测指标、实验结束后处死动物的方法等
Aim of experiment ， Outline of experiments, experimental methods, observational index, executing animal method, et al.

本项目探讨氟砷联合暴露对大鼠学习记忆功能的影响，揭示谷氨酸神经递质及Ⅰ组 mGluRs 介导的信号通路在氟砷联合暴露致学习记忆损害中的分子调控机制。第一部分：初断乳 SD 大鼠随机分组后，饮水途径氟、砷及其联合染毒 3 个月，评价动物的学习记忆功能，麻醉后处死动物，分离海马和大脑皮质组织，检测组织中谷氨酸的含量、mGluRs 介导的 ERK-MAPK 信号通路中关键分子的基因和蛋白表达。第二部分：SD 大鼠按雌:雄为 1:1，交配妊娠后饮水途径进行染毒，至仔鼠出生后 21 天，麻醉后处死仔鼠，分离海马和大脑皮质组织，检测指标同上。

The project is designed to test hypothesis that fluoride and arsenic combined exposure impair the ability of learning and memory,and disturb group Ⅰ mGluRs mediated signal transduction. First, weaned Sprague-Dawley male rats were administrated with drinking water with sodium fluoride, sodium arsenite, both sodium fluoride and sodium arsenite for 3 months. At the end of experiment, rats were anesthetized with 10% chloral hydrate. Samples were collected including blood, brain parts. Glutamate, mRNA and protein expression of some key molecules in signal transduction path mediated by group Ⅰ mGluRs were measured. Second, SD rats mated according to female and male ratio of 2:1.Pregnancy rats were administrated with drinking water until postnatal day 21 of offspring.The pups were scrificate with 10% chloral hydrate. Blood, brain parts were collected. The indexes deteced were same as the part one.</td></tr>
</table>

申请者声明 Announcement of applicant	我将自觉遵守实验动物福利伦理原则，随时接受实验动物伦理委员会的监督与检查，如违反规定，自愿接受处罚。<br>I will abide by the rules of animal experimental ethics, accept the supervision and inspection of the animal experimental ethics committee, and accept the punishment if any infringement.)<br>课题负责人签名：Shoufang Jiang<br>2011年 3月 3日	
审查依据 Inspection contents	1．该项目是否必须用实验动物进行实验，即能否用计算机模拟、细胞培养等非生命方法替代动物或用低等动物替代高等动物进行实验(Does laboratory animal must be used in the project? Could other methods such as computer simulation．cell culture or using the low-grade animal instead of the high－grade animal?)<br>2．表中所填申请人资格和所用动物的品种品系、质量等级、规格是否合适，能否通过改良设计方案或用高质量的动物来减少所用动物的数量(Are the qualification of applicant，species or strain，grade and specifications of animals suitable? Could the quantity of animals be reduced by improving the study design or using high quality animals?)<br>3．能否通过改进实验方法、调整实验观测指标、改良处死动物的方法，来优化实验方案、善待动物(Could the study design and animal treatment be refined by ameliorating experimental method，adjusting observational index．executing animal method?)	
审查结果 Results of inspection	实验动物伦理委员会委员意见<br>Members attitude of the Animal Care Welfare Committee	签名：
	实验动物伦理委员会意见<br>Attitude of the Animal Care Welfare Committee	主任委员签名：<br>河北联合大学实验动物伦理委员会<br>盖章<br>1302032100022<br>2011 年 3 月 3 日
备注:<br>Remark		

说明：

1．编号由实验动物伦理委员会办公室分配并填写。

2．表格所有填写内容请用签字笔填写或电脑打印(签名处除外)。

3．需随本表递交相关审查资料如实验方案、课题标书等。要求写明项目的意义、必要性、项目中有关实验动物的用途、饲养管理或实验处置方法、预期出现的对动物的伤害、处死动物的方法、项目进行中涉及动物福利和伦理问题的详细描述。

4．此表双面打印，一式三份，其中一份留实验动物伦理委员会办公室存档。

本表归档人员签名：　　　　本表归档时间：　　年　　月　　日
